# Supplementary material for: Efficacy and Safety of SGLT-2 Inhibitors for Treatment of Diabetes Mellitus among Kidney Transplant Patients: A Systematic Review and Meta-Analysis
Source: Med Sci (Basel). 2020 Nov 17;8(4):47. doi: 10.3390/medsci8040047 (PMC7712903; doi:10.3390/medsci8040047)
Supplement: Supplementary file 1 [file medsci-08-00047-s001.pdf]

**Supplementary Appendix**

Search strategy

Database(s): EBM Reviews - Cochrane Central Register of Controlled Trials March 2020, EBM Reviews - Cochrane Database of Systematic Reviews 2005 to April 22, 2020, Embase 1974 to 2020 April 29, Ovid MEDLINE(R) and Epub Ahead of Print, In-Process & Other Non-Indexed Citations and Daily 1946 to April 29, 2020

Search Strategy:

| # | Searches | Results |
|---|---|---|
| 1 | exp kidney transplantation/ | 249101 |
| 2 | ((kidney* or renal) adj3 (transplant* or graft* or allotransplant* or "allo-transplant*" or homotransplant* or "homo-transplant*" or retransplant* or "re-transplant*" or allograft* or "allo-graft*" or homograft* or "homo-graft*" or "cadaver kidney*")).ti,ab,hw,kw. | 301190 |
| 3 | 1 or 2 | 301641 |
| 4 | exp sodium glucose cotransporter 2 inhibitor/ | 10188 |
| 5 | (capagliflozin* or dapagliflozin* or empagliflozin* or ertugliflozin* or "SGLT2 inhibitor*" or "SGLT-2 inhibitor*" or "sodium dependent glucose cotransporter 2 inhibitor*" or "sodium glucose cotransporter 2 inhibitor*" or "sodium glucose co-transporter 2 inhibitor*").ti,ab,hw,kw. | 17070 |
| 6 | 4 or 5 | 17778 |
| 7 | 3 and 6 | 131 |
| 8 | (exp animals/ or exp nonhuman/) not exp humans/ | 11053593 |

| | | |
|---|---|---|
| 9 | ((alpaca or alpacas or amphibian or amphibians or animal or animals or antelope or armadillo or armadillos or avian or baboon or baboons or beagle or beagles or bee or bees or bird or birds or bison or bovine or buffalo or buffaloes or buffalos or "c elegans" or "Caenorhabditis elegans" or camel or camels or canine or canines or carp or cats or cattle or chick or chicken or chickens or chicks or chimp or chimpanze or chimpanzees or chimps or cow or cows or "D melanogaster" or "dairy calf" or "dairy calves" or deer or dog or dogs or donkey or donkeys or drosophila or "Drosophila melanogaster" or duck or duckling or ducklings or ducks or equid or equids or equine or equines or feline or felines or ferret or ferrets or finch or finches or fish or flatworm or flatworms or fox or foxes or frog or frogs or "fruit flies" or "fruit fly" or "G mellonella" or "Galleria mellonella" or geese or gerbil or gerbils or goat or goats or goose or gorilla or gorillas or hamster or hamsters or hare or hares or heifer or heifers or horse or horses or insect or insects or jellyfish or kangaroo or kangaroos or kitten or kittens or lagomorph or lagomorphs or lamb or lambs or llama or llamas or macaque or macaques or macaw or macaws or marmoset or marmosets or mice or minipig or minipigs or mink or minks or monkey or monkeys or mouse or mule or mules or nematode or nematodes or octopus or octopuses or orangutan or "orang-utan" or orangutans or "orang-utans" or oxen or parrot or parrots or pig or pigeon or pigeons or piglet or piglets or pigs or porcine or primate or primates or quail or rabbit or rabbits or rat or rats or reptile or reptiles or rodent or rodents or ruminant or ruminants or salmon or sheep or shrimp or slug or slugs or swine or tamarin or tamarins or toad or toads or trout or urchin or urchins or vole or voles or waxworm or waxworms or worm or worms or xenopus or "zebra fish" or zebrafish) not (human or humans or patient or patients)).ti,ab,hw,kw. | 9514641 |

| | | |
|---|---|---|
| 10 | 7 not (8 or 9) | 131 |
| 11 | limit 10 to (editorial or erratum or note or addresses or autobiography or bibliography or biography or blogs or comment or dictionary or directory or interactive tutorial or interview or lectures or legal cases or legislation or news or newspaper article or overall or patient education handout or periodical index or portraits or published erratum or video-audio media or webcasts) [Limit not valid in CCTR,CDSR,Embase,Ovid MEDLINE(R),Ovid MEDLINE(R) Daily Update,Ovid MEDLINE(R) In-Process,Ovid MEDLINE(R) Publisher; records were retained] | 14 |
| 12 | from 11 keep 1 | 1 |
| 13 | (10 not 11) or 12 | 118 |
| 14 | remove duplicates from 13 | 94 |
